# Supplementary material for: The genome sequence of the Thor’s Fritillary, Boloria thore (Hübner, 1803) (Lepidoptera: Nymphalidae)
Source: Wellcome Open Res. 2025 Jul 31;10:401. [Version 1] doi: 10.12688/wellcomeopenres.24638.1 (PMC12739373; doi:10.12688/wellcomeopenres.24638.1)
Supplement: Peer review history

# Reviewer report: Reviewer response for version 1

**DOI:** 10.21956/wellcomeopenres.27152.r142415

**Published:** 2026-01-02

**Authors:** Daubian Santos

**Affiliations:** 1 Universidade Federal do ABC, Santo André, State of São Paulo, Brazil

The manuscript is well written, with a methodology clear and efficient. This work was included in the importnat and relevant Project Psyche for Lepidoptera conservation. My only comment is that, although it is not obligated, the authors may include the reference of species authors original descriptions cited in the text, for example, B. selene and the plants V. palustris and V. canina. The authors use "Boloria thore (Hübner, 1803)" in the title, but it may be also present in teh text. That said, the authors did a very good project.

Are sufficient details of methods and materials provided to allow replication by others?

Yes

Is the rationale for creating the dataset(s) clearly described?

Yes

Are the datasets clearly presented in a useable and accessible format?

Yes

Are the protocols appropriate and is the work technically sound?

Yes

Reviewer Expertise:

Genetic extraction and entomology

I confirm that I have read this submission and believe that I have an appropriate level of expertise to confirm that it is of an acceptable scientific standard.

# Reviewer report: Reviewer response for version 1

**DOI:** 10.21956/wellcomeopenres.27152.r139815

**Published:** 2025-12-24

**Authors:** Jose Ramon Planta

**Affiliations:** 1 University of the Philippines Diliman, Quezon city, Philippines

This report covers a significant portion of the assembly, metrics, and structure of the *B. thore *genome. Here are a few comments that the authors can address and incorporate into this report:

 1. Although implied, please mention that the *B. thore* genome is diploid. Is this the first chromosome-scale assembly of the *B. thore *genome? If so, please indicate this in the manuscript.

 2. In Table 1, information on sequencing coverage/depth can be added.

 3. The BUSCO score can be added and mentioned for haplotype 2 (in Figure 5 and Table 4).

 4. Figures 5 and 6 can also include figures for haplotype 2. 

 5. In Table 4, where applicable, metrics for haplotype 2 can also be included.

Are sufficient details of methods and materials provided to allow replication by others?

Yes

Is the rationale for creating the dataset(s) clearly described?

Yes

Are the datasets clearly presented in a useable and accessible format?

Yes

Are the protocols appropriate and is the work technically sound?

Yes

Reviewer Expertise:

Plant genetics and genomics

I confirm that I have read this submission and believe that I have an appropriate level of expertise to confirm that it is of an acceptable scientific standard.

# Reviewer report: Reviewer response for version 1

**DOI:** 10.21956/wellcomeopenres.27152.r128171

**Published:** 2025-09-05

**Authors:** Quentin Rougemont

**Affiliations:** 1 Université Paris Saclay, Paris, France

**Review of** **'The genome sequence of the Thor’s Fritillary,** ***Boloria thore*** **(Hübner, 1803) (Lepidoptera: Nymphalidae)**

 This is a high-quality genome assembly of a new species *Boloria thore* *. *This will provide valuable ressources for underrepresented non-model species for a wide range of genomic studies and comparative analyses, especially given that the two haplotypes were provided and a W and Z chromosomes assembled. This genome may be usefull for future conservation genomics studies.

 The genome (haplotype1) has 31 chromosomes with a high level of quality based on all the quality metrics provided (Busco, N50, QV, etc).

 The genome was easily accessible online, which is great.

 The whole pipeline follows standard practice and the report of the results meet the quality requirement. The pipeline is also easilly findable online following the provided links.

 The merian plot was really nice, though I am wondering if it is possible to use more contrasted colors to associated each chromosome with the different merian elements?

 Very minor comments:

 It would be nice to specify whether only default parameters were used for each tools or if some parameters had to be set to some specific values.

 Similarly, I found that busco tends to provide different results depending on the tools being used (Augustus, metaeuk, miniprot), the latter providing higher scores for instance, so please specify, which gene mapper was used.

 In the same vein, why not showing busco score from the ‘papilionoidea_odb12’ (I obtained the following when checking the haplotype 1: C:99.4%[S:99.0%,D:0.4%],F:0.0%,M:0.6%,n:6917,E:2.6% (based on miniprot and N= 6917, which is more than lepidoptera).

 All the rest follows standard practices and meet the quality requirement

Are sufficient details of methods and materials provided to allow replication by others?

Yes

Is the rationale for creating the dataset(s) clearly described?

Yes

Are the datasets clearly presented in a useable and accessible format?

Yes

Are the protocols appropriate and is the work technically sound?

Yes

Reviewer Expertise:

population genomics, evolutionary biology

I confirm that I have read this submission and believe that I have an appropriate level of expertise to confirm that it is of an acceptable scientific standard.
